# A Review of Textile Hydrogel Integration in Firefighting Personal Protective Clothing

**DOI:** 10.3390/polym18020204

**Published:** 2026-01-12

**Authors:** Sydney Tindall, Meredith McQuerry, Josephine Bolaji

**Affiliations:** 1 FSU-FAMU College of Engineering, Florida State University, Tallahassee, FL 32306, USA; 2 Therma NOLE Comfort Lab^®^, Jim Moran College of Entrepreneurship, Florida State University, Tallahassee, FL 32306, USA

**Keywords:** hydrogel, firefighting, protective clothing, materials science, textiles, aramids, thermoregulation

## Abstract

Traditional firefighting protective clothing materials, such as meta- and para-aramid fibers, provide significant thermal protection but often fail to adequately manage heat stress and moisture, especially due to the incorporation of semi-permeable membranes within the three-layer garment structure known as turnout gear. Integrating hydrogels into textiles for firefighting personal protective clothing (PPC) could enhance thermoregulation and moisture management, providing firefighters with improved comfort and safety. Hydrogels are three-dimensional, hydrophilic polymer networks capable of holding substantial amounts of water. Their high water content and excellent thermal properties make them ideal for cooling applications. Therefore, this review focuses on the potential of hydrogel-infused textiles to improve firefighters’ PPC by enhancing thermal comfort and moisture management. Specifically, hydrogel structures and engineered properties for enhanced performance are presented, including smart hydrogels and hydration customization mechanisms. Hydrogel integration into firefighting PPC for moisture management and improved thermoregulation is explored, including current and future market projections and state-of-the-art clinical trial findings. Overall, the future of hydrogel-integrated textiles for firefighting PPC is bright, with numerous advancements and trends poised to enhance the safety, comfort, and performance of protective gear.

## 1. Introduction

Firefighters face extreme conditions, including high temperatures, flames, and hazardous environments necessitating highly effective personal protective equipment (PPE) [1]. Traditional PPE materials, such as meta- and para-aramid fibers (e.g., Nomex^®^ and Kevlar^®^), provide significant thermal protection but often fail to adequately manage heat stress and moisture, especially due to the incorporation of semi-permeable membranes in the three-layer garment structure known as turnout gear. This combination of liquid blocking nonwoven materials and multi-layer composites can quickly lead to heat exhaustion and decreased performance for the firefighter [1,2,3]. Integrating hydrogels into textiles for PPE could enhance thermoregulation and moisture management, providing firefighters with improved comfort and safety [2]. Therefore, this review focuses on the potential of hydrogel-infused textiles to improve firefighters’ PPE by enhancing thermal comfort and moisture management.

**Figure 1:**
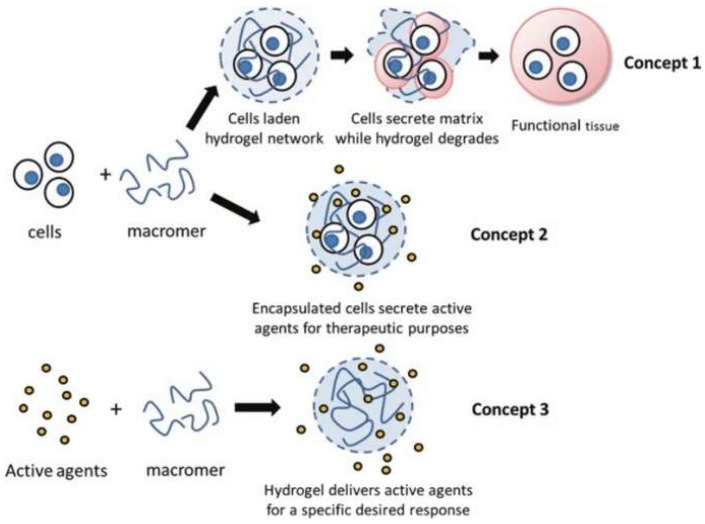
Schematic of the molecular structure of a hydrogel [6]. Reproduced with permission from Khan et al., Polymer Bulletin; published by Springer, 2022.

Hydrogels are three-dimensional, hydrophilic polymer networks capable of holding substantial amounts of water [4,5]. Their high-water content and excellent thermal properties make them ideal for cooling applications [4,5]. When exposed to heat, hydrogels can absorb and retain large amounts of water, releasing it slowly to provide a cooling effect [4]. Figure 1 illustrates the molecular structure of a hydrogel, showing the cross-linked polymer chains that form a network capable of retaining water molecules [6]. This structure allows hydrogels to absorb significant amounts of water, providing their characteristic cooling effect [6].

In addition to cooling properties, hydrogels also offer excellent moisture management [7,8]. They can wick moisture away from the body, keeping the skin dry and reducing the risk of skin irritation, heat rash [8], and potentially, steam burns. Integrating hydrogels into textile fibers could enhance the overall comfort of PPE, making it easier for firefighters to perform their duties under extreme conditions [8].

Firefighters’ PPE typically includes several layers, each serving a specific purpose, such as thermal insulation, flame resistance, and liquid barrier protection [9]. By incorporating hydrogel-infused textiles into these layers, it is possible to enhance their functionality and provide additional benefits [10]. For instance, a moisture barrier layer integrated with hydrogels could more effectively manage sweat and reduce heat stress, while the thermal insulation layer worn closer to the skin could benefit from the cooling properties of hydrogels [9].

Integrating hydrogels into firefighting PPE not only addresses many of the limitations of traditional flame-resistant materials but also introduces innovative solutions to improve the safety and comfort of firefighters [10]. This paper explores the market size, growth, trends, available competitor products, and products in preclinical or clinical stages related to hydrogel-integrated textiles for firefighting PPE.

### 1.1. Hydrogel Structure Properties

Hydrogels are three-dimensional, cross-linked polymer networks that can absorb and retain substantial amounts of water due to the hydrophilic nature of their polymer chains [4]. The polymer matrix is typically composed of hydrophilic groups such as hydroxyl (-OH), carboxyl (-COOH), amide (-CONH-), and sulfate (-SO_3_H) groups, which interact with water molecules through hydrogen bonding [5]. The high-water content within the hydrogel matrix provides not only cooling properties but also soft, flexible mechanical properties that are crucial for comfort in wearable applications like firefighting PPE [4].

The mechanical properties of hydrogels, such as their tensile strength, elasticity, and compressive modulus, can be finely tuned by adjusting the degree of cross-linking and the nature of the polymer network [6]. For instance, covalently cross-linked hydrogels typically exhibit higher mechanical strength but lower flexibility compared to physically cross-linked hydrogels, which rely on non-covalent interactions such as ionic bonds or hydrogen bonds [6]. The ability to modify these properties makes hydrogels highly versatile for integrating into textiles where different mechanical demands may be required for different functionalities across the many layers of the turnout suit (e.g., outer shell, moisture barrier, and thermal liner) [4].

Furthermore, the thermal stability of hydrogels is a key property when considering their application in high-temperature environments like firefighting [9]. Hydrogels composed of polymers such as polyvinyl alcohol (PVA), polyethylene glycol (PEG), or polyacrylamide (PAM) are known for their excellent thermal stability [9]. These polymers do not degrade at the temperatures typically encountered in firefighting scenarios (150–1000 °C), ensuring the hydrogel maintains its integrity and continues to provide cooling even under extreme conditions [10].

Another critical aspect is the diffusion properties of hydrogels, which govern how water and other molecules move through the polymer network [4]. This is particularly important for cooling applications, as the rate of water evaporation from the hydrogel directly influences the cooling effect [4]. The diffusion coefficient can be controlled by altering the polymer composition and cross-linking density, allowing for the design of hydrogels that provide sustained cooling over extended periods [4].

### 1.2. Functionalization of Hydrogels for Enhanced Performance

One of the latest advancements in hydrogel technology is the functionalization of polymer networks to introduce additional properties that enhance the performance of firefighting PPE. This functionalization includes the incorporation of antimicrobial agents, flame retardants, and nanomaterials to improve the safety and durability of the hydrogel.

For instance, the incorporation of silver nanoparticles into hydrogels has been shown to provide significant antimicrobial properties [11]. These silver nanoparticle-infused hydrogels can be highly effective in preventing bacterial infections, which is crucial for firefighting gear that may come into contact with burns, cuts, scrapes, or other open wounds [11]. The antimicrobial action of silver nanoparticles is well-documented, as they can disrupt microbial cell membranes and inhibit the replication of bacteria, making them ideal for use in PPE where hygiene and safety are paramount [11].

In addition to antimicrobial properties, hydrogels can be functionalized with flame-retardant chemicals such as phosphorus-containing compounds. These compounds enhance the hydrogel’s fire resistance by forming a protective char layer when exposed to high temperatures, which helps to prevent the spread of flames [12]. Phosphorus-based flame retardants are particularly effective because they act both in the condensed phase (by promoting char formation) and in the gas phase (by releasing flame-inhibiting species) [12].

## 2. Customization of Hydrogel Mechanical Properties

The mechanical properties of hydrogels, such as their stiffness, toughness, and elasticity, are critical for their application in firefighter turnout gear. These properties can be customized through techniques such as double-network hydrogels or interpenetrating polymer networks (IPNs). Double-network hydrogels are engineered by combining two interpenetrating polymer networks with different mechanical characteristics. The first network is typically rigid and provides structural integrity, while the second network is softer and more flexible, offering elasticity and resistance to mechanical stress [13]. This combination is particularly useful in PPE, where materials must be both durable and comfortable to wear [13].

Interpenetrating polymer networks (IPNs) are another approach to enhance the mechanical properties of hydrogels. IPNs consist of two or more polymer networks that are physically interlocked but not covalently bonded, providing increased toughness and resilience [14]. These networks can improve the durability of hydrogels under mechanical stress, such as the bending and stretching that occur during firefighting activities [14]. IPNs are particularly advantageous because they allow for greater control over the swelling behavior and mechanical strength of the hydrogel [14].

### 2.1. Smart Hydrogels for Responsive PPE

Smart hydrogels represent a significant potential design innovation. These hydrogels can respond to environmental stimuli such as temperature, pH, or mechanical force, offering dynamic protection that adapts to the conditions encountered during firefighting. Thermo-responsive hydrogels, for example, can change their swelling behavior in response to temperature fluctuations. At lower temperatures, these hydrogels remain hydrated and provide a cooling effect [15]. However, as temperatures rise, they undergo a phase transition that causes them to expel water, enhancing evaporative cooling precisely when it is needed most [15]. This temperature-responsive behavior is critical in firefighting, where rapid changes in environmental conditions are common [15,16].

Moreover, pH-responsive hydrogels can be used in PPE to deliver therapeutic agents in response to changes in skin pH, which can occur due to sweating or exposure to chemicals. These hydrogels can release anti-inflammatory or antimicrobial agents when a shift in pH is detected, providing immediate protection and treatment for the wearer [16]. Such smart hydrogels could be particularly useful in treating injuries on the field without needing to remove the PPE [16]. In this case, application to the next-to-skin thermal liner facecloth layer, or even station wear and base layers, could be considered.

### 2.2. Hydration and Dehydration Mechanism

The hydration and dehydration cycles of hydrogels are central to their function, especially for the application of firefighting protective clothing [4]. When a hydrogel is exposed to a high-temperature environment, such as during firefighting, the water molecules absorbed within the hydrogel matrix begin to evaporate [4]. This phase change from liquid to vapor requires significant energy, which is absorbed from the surrounding environment, thereby producing a cooling effect [4]. The efficiency of this cooling mechanism depends on several factors, including the water content of the hydrogel, the ambient temperature, and the relative humidity [4].

Hydrogels can be engineered to optimize these cycles for maximum cooling efficiency [5]. For example, superabsorbent polymers (SAPs) like sodium polyacrylate can absorb hundreds of times their weight in water, which can then be gradually released during dehydration [5]. By fine-tuning the cross-linking density and polymer composition, it is possible to control the rate at which water is released, ensuring that the cooling effect is both immediate and sustained [5].

Additionally, the incorporation of hygroscopic materials, such as calcium chloride or magnesium sulfate, into the hydrogel matrix can enhance its water retention capacity [5]. These materials attract and hold water molecules, reducing the rate of evaporation and extending the duration of the cooling effect [5]. This is particularly beneficial in environments where prolonged exposure to high temperatures is expected, as it ensures that the hydrogel remains effective throughout the duration of the firefighting operation [4,5].

Moreover, the physical structure of the hydrogel can be engineered to promote efficient water distribution and evaporation [5,6]. For instance, hydrogels with a porous structure or those incorporating microchannels can facilitate rapid water movement to the surface, enhancing the cooling effect [5]. This structural engineering is crucial for maintaining consistent cooling performance, especially in the dynamic conditions experienced by firefighters [5].

## 3. Integration into Textile Fibers

Integrating hydrogels into textile fibers requires sophisticated manufacturing techniques that ensure the hydrogel is uniformly distributed and securely bonded to the fiber matrix [6,7]. One advanced method is electrospinning, which allows for the fabrication of nanofibers embedded with hydrogel particles [6]. Electrospinning involves the use of a high-voltage electric field to draw a polymer solution into fine fibers, which can then be collected on a substrate to form a nonwoven fabric [6]. By incorporating hydrogel particles into the polymer solution before electrospinning, it is possible to create a composite fiber with embedded hydrogel, providing both moisture management and cooling properties [6,7,8].

In situ polymerization is another method used to integrate hydrogels into textiles [10]. This process involves polymerizing the hydrogel directly within the fiber matrix, which can be achieved through various chemical reactions, such as free-radical polymerization or condensation polymerization [10]. In this approach, monomers and cross-linking agents are infused into the fiber matrix, followed by the initiation of the polymerization reaction, leading to the formation of a hydrogel network within the fibers [8,10]. This method ensures that the hydrogel is firmly anchored within the textile structure, providing long-term durability and consistent performance under the physical-mechanical stresses the material will encounter during firefighting [10].

Coating techniques, such as dip-coating or layer-by-layer assembly, can also be used to apply a thin hydrogel layer onto the surface of textile fibers [7]. Dip-coating involves immersing the textile in a hydrogel solution, allowing the fibers to absorb the solution before being removed and dried, leaving a uniform hydrogel coating [7,17]. Layer-by-layer assembly, on the other hand, involves sequentially dipping the textile in solutions of oppositely charged polyelectrolytes, building up a multilayer hydrogel coating [7]. These coatings can be tailored to achieve specific thicknesses and mechanical properties, depending on the requirements of the PPE [17].

The mechanical integration of hydrogels into textiles also involves ensuring that the hydrogel does not compromise the flexibility and breathability of the fabric [6]. Advanced textile engineering techniques, such as the use of composite yarns or fabrics, can help achieve this balance [4,6]. For instance, hydrogel-infused yarns can be combined with traditional fibers like aramids to create a composite fabric that offers both inherent flame resistance and moisture management [6].

### 3.1. Moisture Management and Thermoregulation

The incorporation of hydrogels into firefighting PPE may significantly enhance moisture management by providing a dual function: absorbing sweat from the skin and providing a cooling effect via evaporation [5]. Hydrogels’ hydrophilic nature allows them to effectively wick moisture away from the body, reducing the risk of overheating and subsequent heat-related injuries such as heat exhaustion or heat stroke [3]. The hydrogel’s ability to hold large amounts of water means that it can act as a reservoir for moisture, gradually releasing it over time to maintain a consistent cooling effect [4]. This is particularly important in firefighting scenarios, where maintaining a stable body temperature is crucial for performance and safety [4,5]. The slow release of water from the hydrogel matrix could ensure that the cooling effect is sustained over extended periods, potentially delaying the onset of incompensable heat loss [4].

In addition to their cooling properties, hydrogels also provide an insulating effect by trapping a layer of water within their polymer network [4,6]. This water layer acts as a thermal buffer, absorbing heat from the external environment and slowing its transfer to the skin [4]. This insulative effect is particularly useful when combined with other PPE layers, such as thermal barriers or flame-resistant fabrics, enhancing the overall protective performance of the gear [4]. Moreover, the integration of hydrogels into the moisture barrier layer of the firefighting composite could prevent the buildup of sweat within the suit, reducing the risk of steam burns [9]. Steam burns occur when sweat trapped inside the turnout suit is rapidly heated by external heat and flame, causing it to vaporize and scald the skin [9]. By wicking sweat away from the skin and dispersing it through the hydrogel matrix, the risk of such injuries may be significantly reduced [9].

### 3.2. Hydrogel Mechanisms to Improve Firefighter Cooling

Firefighters continue to face serious heat-related conditions including heat stress, exhaustion, stroke, and even death, highlighting the urgent need for effective cooling during and immediately after their shifts. Hydrogels embedded in firefighter turnout suits can help reduce heat exhaustion through evaporative cooling, a process that draws heat away from the body by leveraging the gel’s high- water content and strong cross-linked hydrophilic polymer network, which requires a large amount of energy to evaporate. Incorporating hydrogel layers into firefighter protective clothing represents a promising strategy.

When embedded in a fabric laminate, the hydrogel absorbs heat from the wearer and evaporates it to the ambient environment, thereby absorbing latent heat and keeping the skin-facing layers at or below 100 °C until dehydration has occurred [18]. Studies have shown that hydrogel fabric laminates use their super-hydrophilic gel as an effective thermal buffer, markedly enhancing heat resistance and providing insulation from extreme external temperatures [19,20]. One concern with hydrogels is their durability and length of effectiveness. However, research indicates that their robust polymer network should potentially allow hydrogels to retain structural integrity even when saturated, thereby reducing the likelihood of premature failure under high thermal stresses [18,19,21].

From a physiological comfort perspective, hydrogel-based cooling reduces cardiovascular and thermoregulatory strain by slowing the rise in skin and core temperatures during exertion [18]. Conventional cooling vests made of ice- or phase-change-materials have shown some benefit. However, they can be heavy, rigid, or limited in duration [19,21]. By comparison, hydrogels combine high heat-storage capacity and flexibility to passively draw away heat without the excessive added weight or stiffness, potentially reducing heat exhaustion risk and improving safety under extreme thermal conditions [20,21].

Overall, research on integrating hydrogels into firefighter protective clothing is still exploratory but rapidly advancing. Current studies demonstrate clear potential for hydrogels to serve as effective passive cooling layers, yet most evidence comes from laboratory-scale material tests or adjacent applications [18,19,21]. To move toward real-world deployment, future work will need to evaluate long-term durability, dehydration rates under realistic firefighting conditions, garment integration strategies, and the balance between moisture gain and cooling effectiveness. As these technologies mature, hydrogels could become a key component in next-generation turnout gear aimed at reducing heat stress and improving firefighter safety on the job.

## 4. Challenges and Future Developments

Despite the promising benefits of hydrogel integration in firefighting PPE, several challenges must be addressed to optimize their performance and durability [21]. One of the primary challenges is ensuring the hydrogels maintain their functional properties under the extreme conditions encountered in firefighting, such as exposure to open flames, high temperatures, and abrasive surfaces [21]. Research is ongoing to develop hydrogels with enhanced mechanical strength and thermal resistance, which could significantly improve the longevity and safety of hydrogel-infused PPE [21]. One approach involves the incorporation of reinforcing agents, such as nanoparticles or carbon nanotubes, into the hydrogel matrix [21]. These materials could significantly enhance the mechanical properties of the hydrogel, making it more resistant to tearing, compression, and other forms of mechanical stress [21].

Another area of development is the creation of smart hydrogels that can respond to environmental stimuli, such as temperature, pH, or mechanical stress [6]. For example, thermo-responsive hydrogels could be engineered to enhance their cooling effect as the ambient temperature increases, providing dynamic protection tailored to the intensity of the heat exposure These smart hydrogels could be applied to the outer shell layer of the turnout suit base composite, providing real-time adaptation to changing environmental conditions [6].

In addition, the development of eco-friendly, biodegradable hydrogels is gaining attention as the demand for sustainable materials continues to grow [7,22,23]. Biodegradable hydrogels, made from natural polymers such as chitosan, alginate, or cellulose, could provide the same functional benefits as synthetic hydrogels while reducing the environmental impact of laundering and end-of-life disposal [7]. Further, some firefighting experiments have shown that a ternary hybrid hydrogel consisting of carrageenan, vermiculite, and dimethyl methyl phosphate (DMMP) could rapidly reduce combustion temperatures, providing superior fire extinguishment efficiency as a cost-effective bio-based hydrogel [22]. These materials are not only sustainable but also biocompatible, making them suitable for applications where direct contact with the skin is required [7].

The future of hydrogel-integrated firefighting PPE lies in the combination of these advanced materials and smart technologies [10]. By continuing to refine the properties of hydrogels and exploring new methods of integration, it will be possible to create PPE that offers unparalleled protection, comfort, and sustainability, ensuring that firefighters can perform their duties safely and effectively in even the most extreme conditions [8,10]. In order to determine the path forward, the current state of the market and hydrogel technology must first be explored.

### 4.1. Market Growth and Product Analysis

The global hydrogel market is experiencing robust growth. In 2024, the market size was projected to reach approximately USD 29.68 billion and is expected to grow at a compound annual growth rate (CAGR) of 6.6% to reach USD 46.43 billion by 2031 [24,25,26]. This growth is driven by the increasing demand for advanced materials that offer better thermoregulation and moisture management, which are critical for firefighting PPE [24,25,26]. In the United States, the PPE market is projected to grow significantly due to stringent government regulations on workplace safety and increased awareness regarding occupational hazards [26,27]. The US PPE market alone was valued at around USD 14 billion in 2024 and is expected to grow at a CAGR of 11.45% to reach USD 31 billion by 2029 [7,27,28]. Similarly, the European market for hydrogel-integrated PPE textiles is also expanding, driven by similar safety regulations and innovations in textile technologies [24,25,26].

The Asia-Pacific region is emerging as the fastest-growing market for hydrogels. Countries like China, India, Japan, and South Korea are rapidly expanding their healthcare infrastructures and witnessing increased disposable incomes. This boosts the consumption of advanced wound care and medical devices [26,29]. This region also offers attractive production environments due to lower costs of standing up manufacturing facilities and an abundantly skilled workforce [26,29]. Several large North American and European companies have established production bases in Asia to cater to the growing local demand as well as to export to other markets [26].

In summary, there are several notable trends driving the growth of the hydrogel market regarding PPE applications. Enhanced thermal management properties of hydrogels are becoming increasingly popular, providing effective heat dissipation and cooling effects [24,30]. Sustainability is another significant trend, with a growing focus on eco-friendly materials. Hydrogels, which are often derived from natural polymers, align well with this trend [31]. Additionally, technological advancements in hydrogel formulations and textile manufacturing techniques are leading to the development of more efficient and multifunctional PPE [25,26].

Several companies are pioneering the integration of advanced materials in textiles for PPE in the US and Europe, although none have incorporated hydrogels just yet. When comparing various competitor products on the structural firefighting market, some companies like DuPont^®^ focus on thermoregulation and durability, particularly in high-temperature environments, and are known for their renowned protective fabrics like Nomex^®^ and Kevlar^®^, which provide excellent thermal protection and durability [32]. These materials are designed to withstand and dissipate high levels of heat, making them particularly beneficial for firefighters exposed to extreme temperatures. Although they do not currently integrate hydrogel technology, DuPont’s products are known for their reliability under the intense physical demands of firefighting. Incorporating hydrogels into Nomex^®^ and Kevlar^®^ blends, and other fibers used in structural turnout gear such as Polybenzimidazole (PBI), could further enhance thermoregulation by maintaining a safer body temperature and preventing heat-related injuries through their cooling properties.

Some PPE manufacturers also emphasize moisture management and cooling in their PPE solutions [33]. This type of advanced turnout gear, such as the Morning Pride^®^ Pro Fit Pants, integrate features that wick away sweat and provide a cooling effect, which is crucial for reducing the risk of heat stress during firefighting operations [33]. This focus on moisture management by many manufacturers on the market today ensures that firefighters remain comfortable and dry, improving their performance and safety in high-stress environments. Honeywell has also developed other advanced features like toxin-resistant hoods, but there is no evidence that their products incorporate hydrogel layers. By integrating hydrogels, Honeywell and other PPE manufacturers could further enhance cooling and moisture-wicking capabilities, providing better comfort and reducing heat stress more efficiently.

Other flame-resistant materials on the market that stand out include Lenzing’s fibers for their combined thermal and moisture management properties, making them highly versatile [33]. Lenzing FR^®^ textiles offer both flame resistance and enhanced moisture-wicking capabilities, ensuring that firefighters remain cool and dry even in the most challenging environments [25]. However, similar to DuPont^®^ and Honeywell, Lenzing does not currently integrate hydrogels into these materials. The addition of hydrogels to Lenzing’s fiber offerings could provide a dual benefit of superior cooling and moisture management, making the gear more effective in extreme conditions.

Although none of these companies currently leverage the unique properties of hydrogels for integration into the final product of firefighting PPE, incorporating this technology could significantly enhance the end use performance. Hydrogels could improve thermoregulation, provide better cooling and moisture management, and enhance overall comfort and safety for firefighters [24,26,29].

### 4.2. Ongoing Clinical Trials for Commercial Hydrogels

While direct clinical trials on integrating hydrogels into firefighter PPE are limited, the observed benefits of hydrogels in medical and other applications suggest significant potential for enhancing firefighter gear. There are various clinical trials that highlight the broader applications and ongoing research in hydrogel technology, demonstrating their effectiveness in diverse medical and protective applications.

One significant clinical trial, “Effect of the negative pressure therapy dressing compared with hydrogel dressing in the treatment of chronic leg wounds,” investigates the use of hydrogel dressings to manage moisture and promote healing in chronic leg wounds [34]. This study demonstrates how hydrogels can effectively manage moisture, which is highly relevant for improving firefighting PPE by keeping the wearer dry and comfortable under extreme conditions [34]. This trial demonstrates the potential of hydrogels to effectively manage moisture, by keeping the wearer dry and comfortable under extreme conditions [34].

Another clinical trial, “Safety and efficacy study of a hydrogel, applied following removal of myomas,” explores the use of hydrogels in surgical settings to enhance healing and reduce complications [35]. The success of hydrogels in surgical applications underscores their potential in enhancing the protective qualities of firefighting PPE, especially in terms of wound care and recovery from injuries sustained during firefighting operations [35].

Furthermore, the trial “Hydrogel coating to reduce post-surgical infection after joint arthroplasty” examines the effectiveness of hydrogel coatings in preventing post-surgical infections [36]. This research highlights the antimicrobial properties of hydrogels, which could be leveraged in firefighting turnout gear to protect against infections and contaminants that firefighters might encounter in hazardous environments [36].

**Figure 2:**
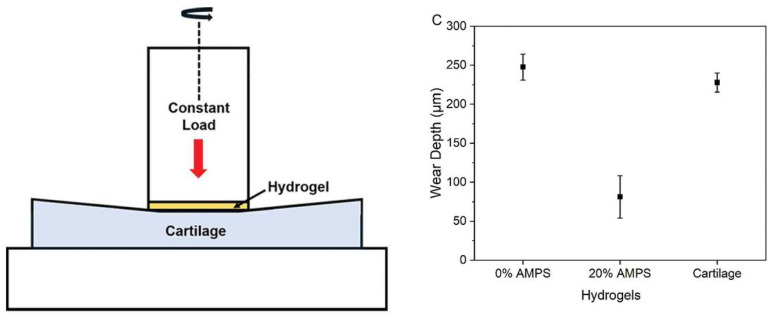
Schematics of how the wear of cartilage versus hydrogels was measured and the wear depth of cartilage and hydrogel samples after 106 cycles under 1 MPa of pressure [40]. Reproduced with permission from Zhao et al., Advanced Functional Materials; published by Wiley, 2022.

A noteworthy ongoing trial at Duke University focuses on a synthetic cartilage hydrogel designed to replace damaged cartilage in arthritic knees [37,38,39]. This hydrogel demonstrates remarkable strength and resilience, outperforming natural cartilage (Figure 2) [37,40]. Its development could provide insights into creating more durable and effective hydrogels for firefighting applications. The synthetic cartilage hydrogel created by the Duke team is made from thin sheets of cellulose fibers laced with polyvinyl alcohol [37,38]. The Duke trial’s findings suggest that the synthetic hydrogel can handle extreme physical stress and provide long-lasting protection, which is essential for firefighting PPE that must endure high temperatures and physical impacts. By incorporating a similar hydrogel into turnout gear, manufacturers could enhance the durability and effectiveness of the PPE, ensuring it can withstand the harsh conditions firefighters face. The hydrogel’s superior strength and resilience also means it could maintain its protective qualities over extended periods, reducing the need for frequent replacements and improving overall safety for firefighters.

In addition, AlgoTx, a clinical-stage biotechnology company, is developing ATX01, a topical hydrogel formulation of amitriptyline [41]. This product targets specific nociceptive sodium channels involved in pain signaling [41]. Currently, ATX01 is in Phase II clinical trials for conditions such as chemotherapy-induced peripheral neuropathy (CIPN) and erythromelalgia (EM), with trials being conducted in both the US and Europe [41]. AlgoTx’s innovative approach focuses on delivering high concentrations of amitriptyline directly to the site of pain, minimizing systemic exposure and reducing potential side effects. The topical hydrogel formulation allows for easy application and sustained drug release, which could be adapted for use in firefighting PPE as a next-to-skin application for pain management from injuries and chronic conditions without impairing mobility or function.

These ongoing clinical trials and preclinical developments are crucial for advancing the safety and efficacy of PPE. Their efforts are critical in addressing the limitations of traditional PPE and providing enhanced protection for those working in extreme conditions. They highlight the potential for significant improvements in firefighter safety and comfort through the integration of hydrogel technology, promising a new generation of protective gear that addresses the limitations of current materials and provides enhanced functionality.

## 5. Future of Hydrogels in Firefighting PPE

The future of hydrogel-integrated textiles for personal protective equipment looks promising, driven by continuous advancements in material science, increasing safety standards, and growing awareness about the importance of firefighter health and safety. As research and development in this field progresses, several trends and innovations are expected to shape the industry. One of the most significant factors influencing the future of hydrogel-integrated textiles is ongoing technological advancements. Innovations in polymer science and nanotechnology are leading to the development of hydrogels with enhanced properties such as increased thermal stability, improved moisture management, and better mechanical strength. These advancements are expected to result in more efficient and multifunctional PPE that can provide superior protection and comfort for firefighters [24,26,30].

As the demand for sustainable and eco-friendly materials grows, the industry is likely to focus more on developing hydrogels derived from natural and biodegradable polymers. This shift towards sustainability not only addresses environmental concerns but also meets the rising consumer demand for green products. Sustainable hydrogels can be integrated into textiles to create PPE that is not only effective in protecting firefighters but also environmentally friendly [17,31]. Future developments in hydrogel technology are expected to allow for greater customization and personalization of PPE. With advancements in 3D printing and smart textiles, it will become possible to create bespoke protective gear tailored to the specific needs and measurements of individual firefighters. This personalized approach will enhance the fit, comfort, and overall effectiveness of the PPE, ensuring that each firefighter has access to the best possible protection [21,25].

The incorporation of smart features into hydrogel-integrated textiles is another exciting development on the horizon. These smart textiles can be embedded with sensors and other electronic components to monitor the health and well-being of firefighters in real-time. For example, sensors can track vital signs, detect hazardous environmental conditions, and provide alerts to prevent heat stress or other injuries. The integration of such smart features will significantly enhance the safety and efficiency of firefighters during operations [25,26]. As the industry evolves, regulatory bodies are expected to implement stricter safety standards for firefighter PPE. These regulations will likely drive the adoption of advanced hydrogel-integrated textiles that meet higher performance criteria. Compliance with these standards will ensure that firefighters are equipped with the most effective and reliable protective gear available, further enhancing their safety and operational capabilities [17,24].

The market for hydrogel-integrated textiles in PPE is projected to grow significantly over the coming years. Increasing investment in research and development, coupled with rising awareness about the benefits of advanced PPE, will drive market expansion. Companies are likely to invest more in developing innovative products and bringing them to the market, resulting in a broader range of high-performance PPE options for firefighters [24,26,28]. In conclusion, the future of hydrogel-integrated textiles for firefighting PPE is bright, with numerous advancements and trends poised to enhance the safety, comfort, and performance of protective gear. As technology continues to evolve, the industry will see the introduction of more sophisticated and sustainable materials, personalized protective solutions, and smart features that collectively contribute to the well-being and efficiency of firefighters. These developments will not only address the current limitations of traditional PPE but also pave the way for a new era of innovation and excellence in firefighter safety.

## Data Availability

No new data were created or analyzed in this study.
